# Supplementary material for: An integrative method to decode regulatory logics in gene transcription
Source: Nat Commun. 2017 Oct 19;8:1044. doi: 10.1038/s41467-017-01193-0 (PMC5715098; doi:10.1038/s41467-017-01193-0)
Supplement: Supplementary file 1 — Supplementary Information [file 41467_2017_1193_MOESM1_ESM.pdf]

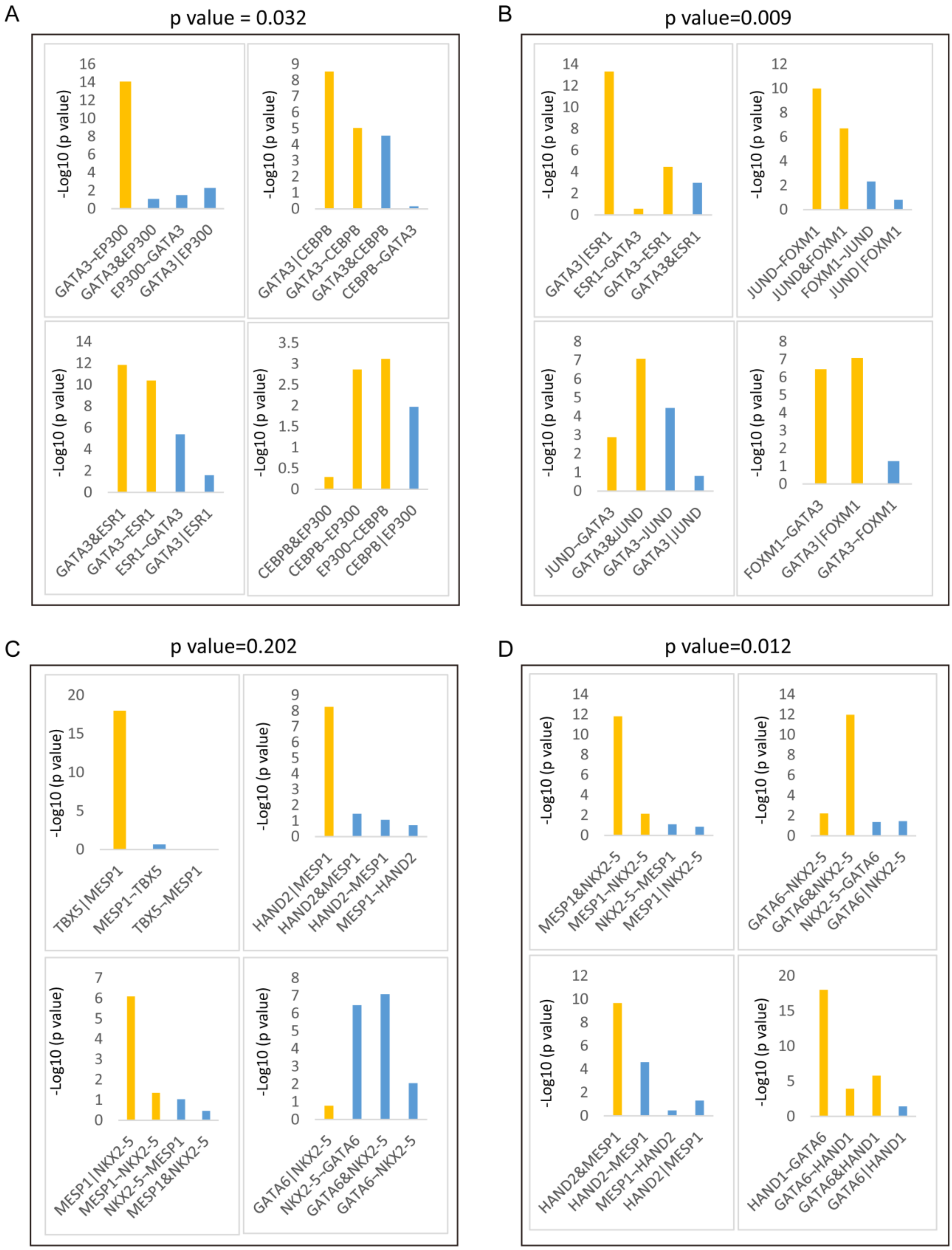

Supplementary Figure 1. Comparison of TF logic target genes between the enriched URLs and the Negative control. A (T1-T2 stage) and B (T2-T3 stage) are human breast cancer data, C (T1-T2 stage) and D (T2-T3 stage) are hiPSC-CM data. Orange and blue bars represent enriched URLs and "Negative control", respectively.

A: T1-T2 stage

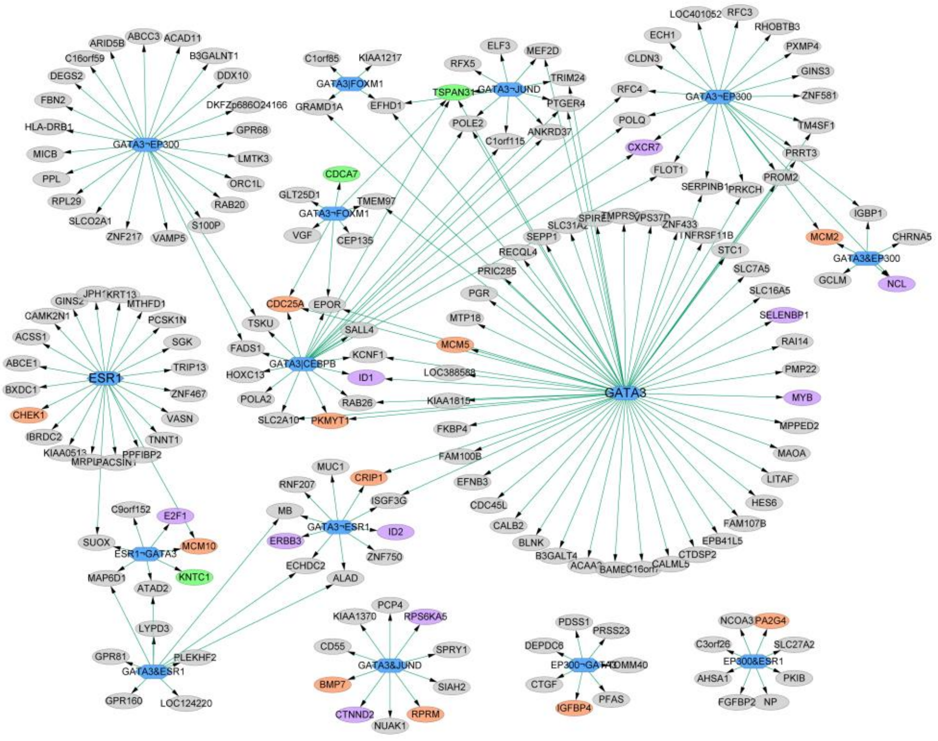

B: T2-T3 stage

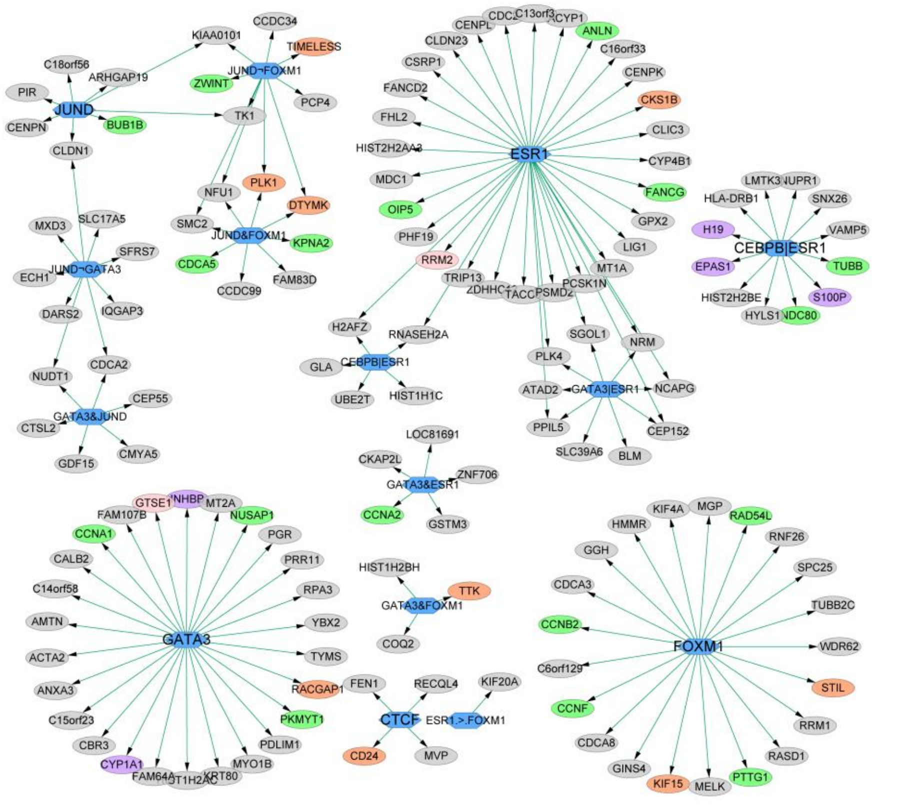

Supplementary Figure 2. E2-responsible TRNs in breast cancer. For A (at T1-T2 stage) and B (at T2-T3 stage), hexagon nodes in blue are TF logics. Oval nodes refer to target genes, in red (apoptosis and p53 pathways), in green (cell cycle), in purple (cell proliferation), in orange (other cancer related pathways) and in gray (others). Logics include AND (&), NOT (¬) and OR (|).

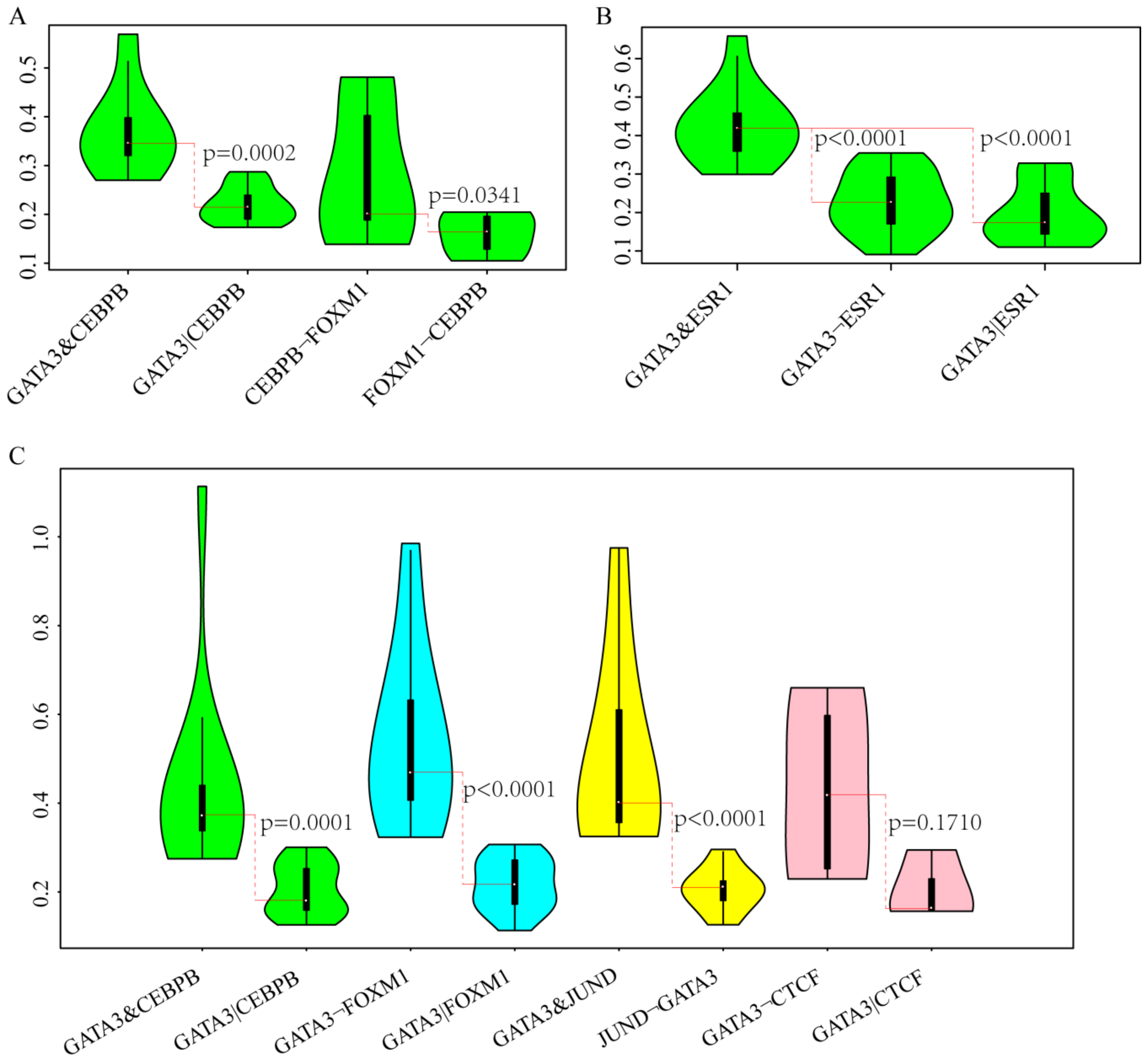

Supplementary Figure 3. Validation of predicted logics in breast cancer cell using gene expression data obtained after TF knockdown. Violin plot shows the density distribution of target genes controlled by a TF logic due to TF knockdown. A, knockdown of CEBPB. B, knockdown of ESR1. C, knockdown of GATA3. p value is calculated by ANOVA and corrected by False Discovery Rate.

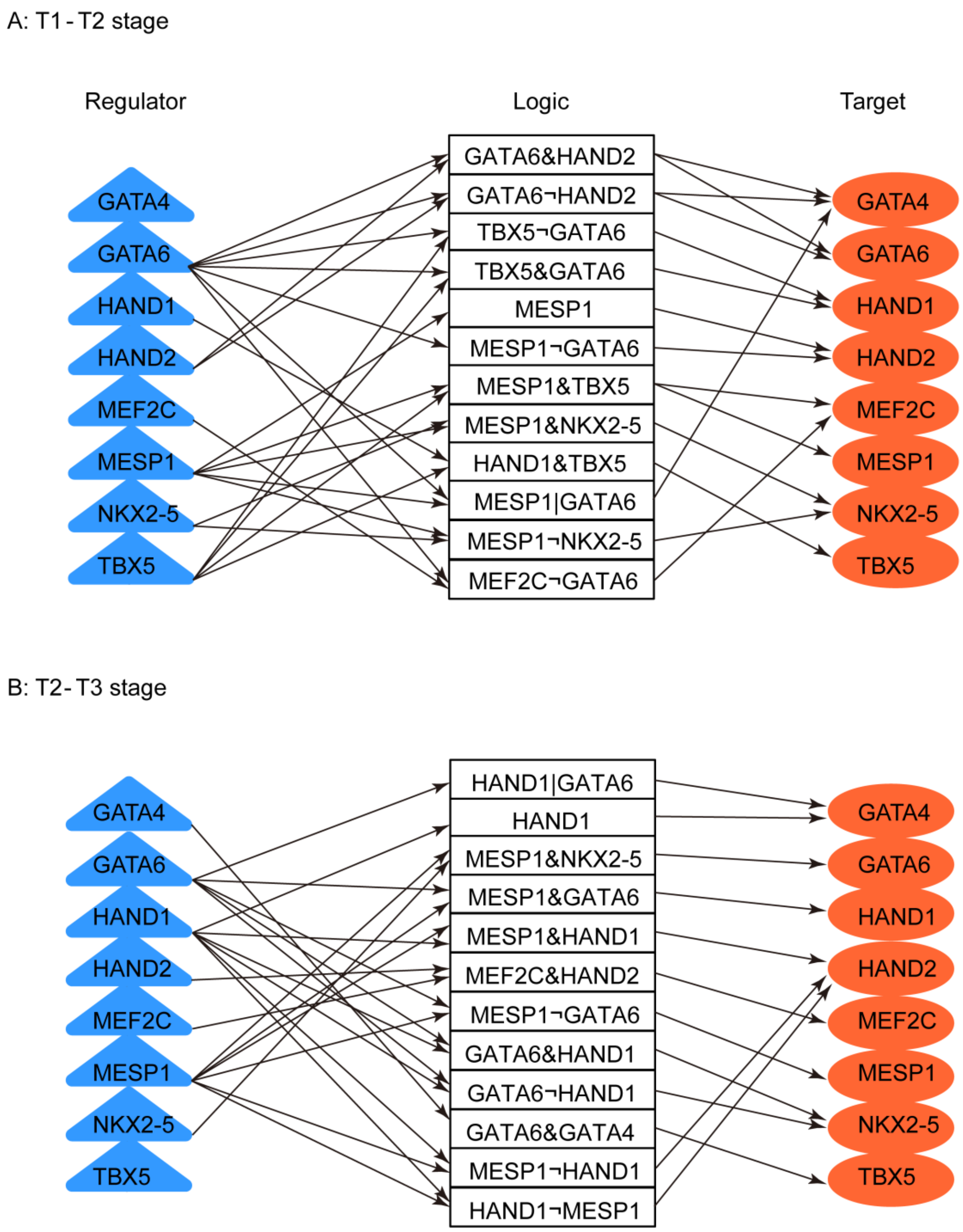

Supplementary Figure 4. Regulatory logics formed by 8 TFs during the hiPSC-derived CM differentiation at T1-T2 stage (A) and T2-T3 stage (B). Logics including AND (&), NOT (¬) and OR (|).

A: T1-T2 stage

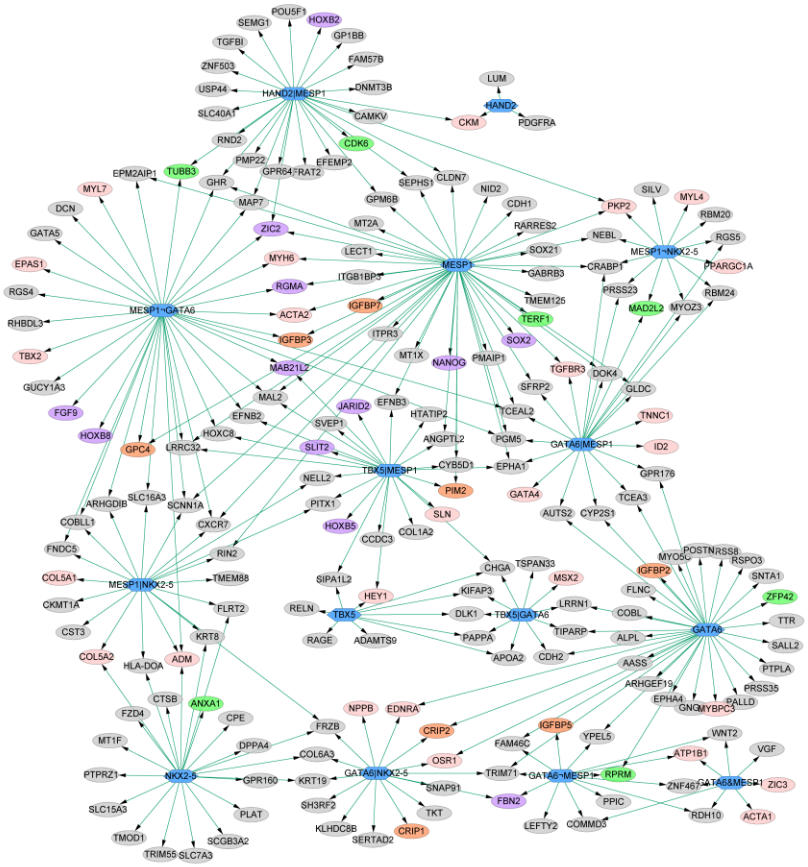

B: T2-T3 stage

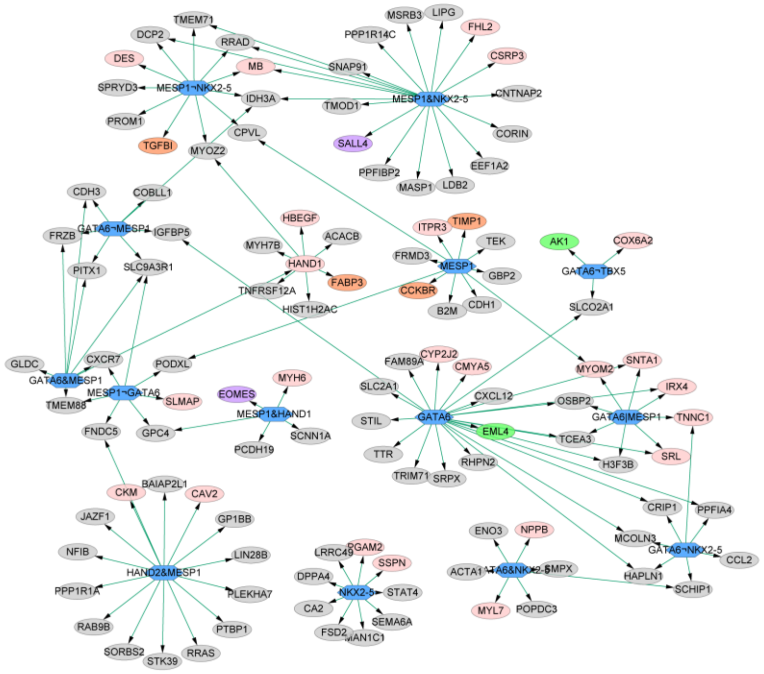

Supplementary Figure 5. The TRNs of hiPSC-derived CM differentiation. For A (at T1-T2 stage) and B (at T2-T3 stage), hexagon nodes in blue are TF logics. Oval nodes refer to target genes, in red (heart development and function), in green (cell cycle), in purple (cell proliferation), in orange (embryonic development) and in gray (others). Logics include AND (&), NOT (¬) and OR (|).

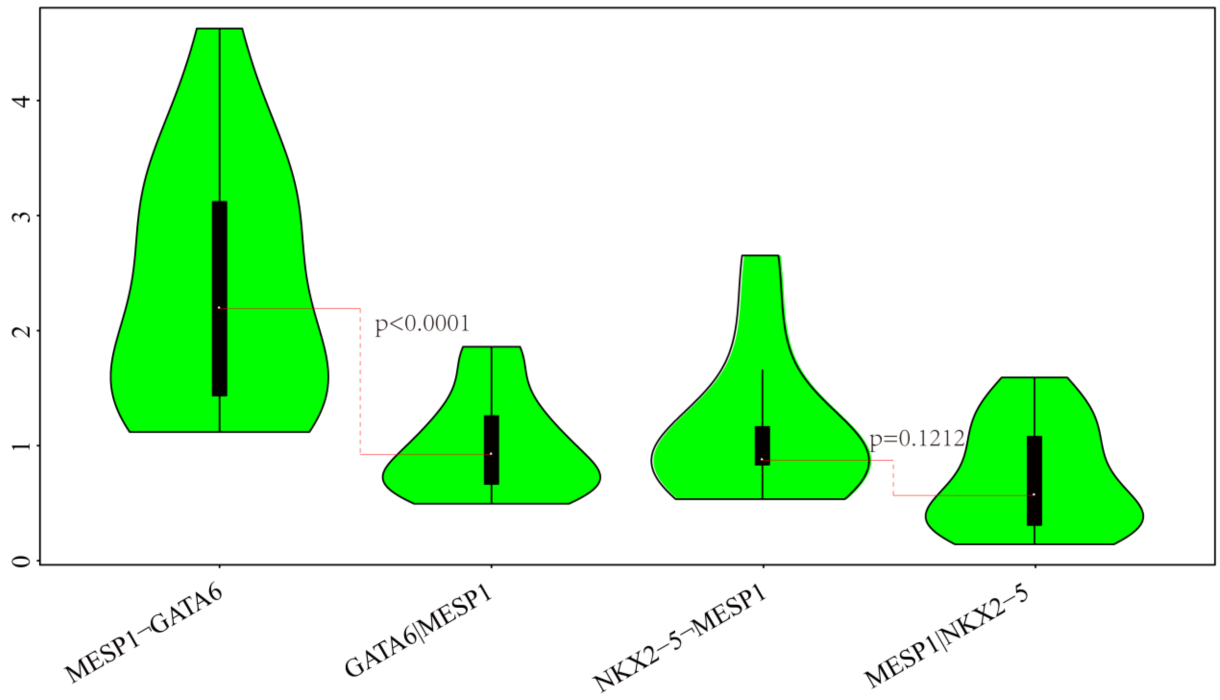

Supplementary Figure 6. Validation of predicted logics in mouse using gene expression data obtained after Mesp1 overexpression. Violin plot show the fold change of density distribution of target genes controlled by a TF logic due to Mesp1 overexpression. p value is calculated by ANOVA and corrected by False Discovery Rate.

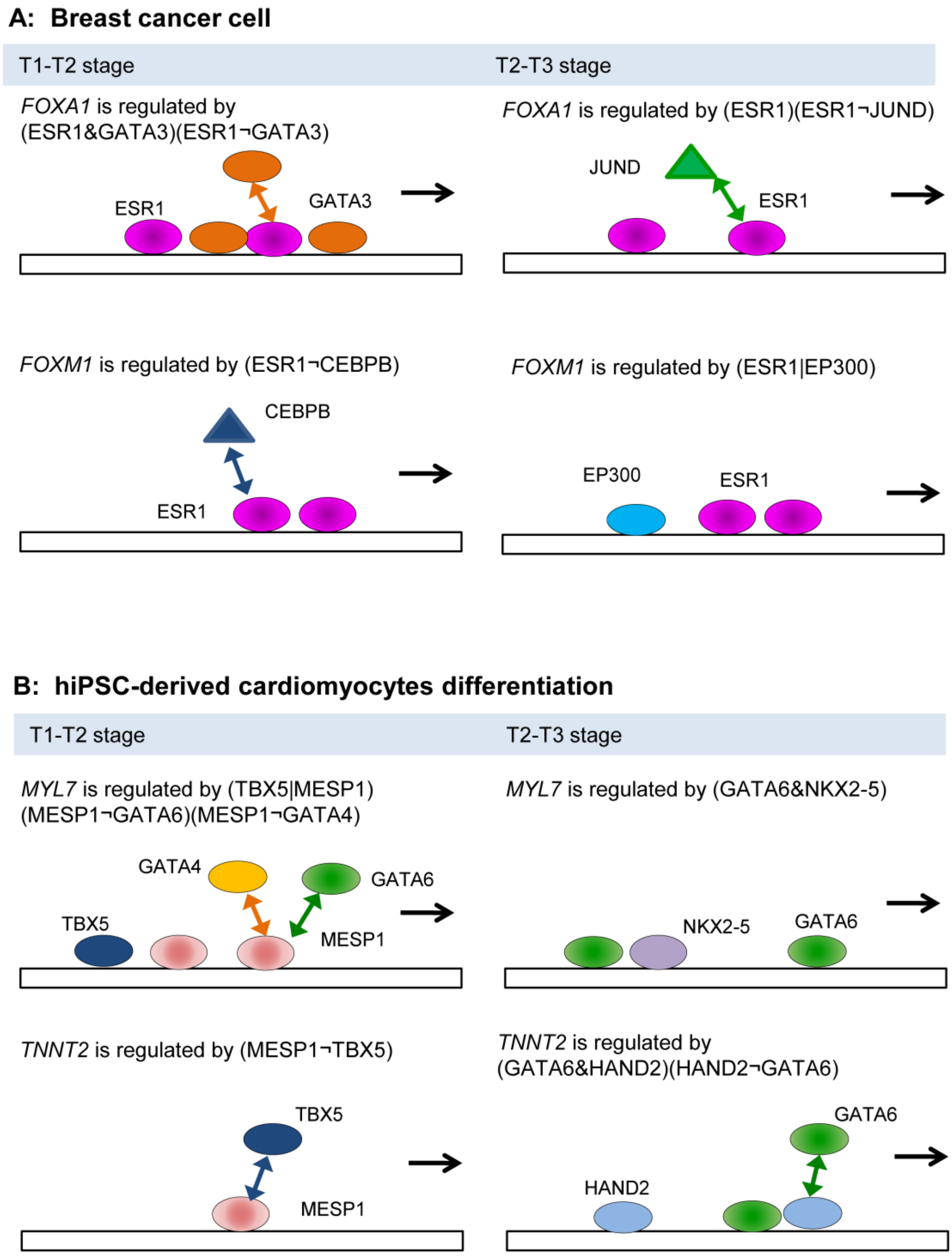

Supplementary Figure 7. Example genes regulated by TF logics. A, E2-induced response of breast cancer. B, hiPSC derived CM development. Regulatory logic relations are represented as: AND (&), NOT (¬) and OR (|).

Supplementary Table 1. URLs of two TFs and their probabilities

URLs	A	B	A | B	A & B	A ¬ B	B ¬ A
Probabilities	ω_1	ω_2	ω_3	ω_4	ω_5	ω_6

Supplementary Table 2. Composite variables of two TFs and their model coefficients

Composite variables	Y_A	Y_B	$Y_A Y_B$	Y_A^2	Y_B^2	$Y_A Y_B^2$	$Y_A^2 Y_B$	$Y_A^2 Y_B^2$
Coefficients	β_1	β_2	β_3	β_4	β_5	β_6	β_7	β_8

Supplementary Table 3. The model coefficients are functions of the kinetic parameters and URLs' probabilities

$\beta_1 = (\omega_1 + \omega_3/2 + \omega_5)k_A$	$\beta_2 = (\omega_1 + \omega_3/2 + \omega_6)k_B$	$\beta_3 = -(\omega_3/2 + \omega_5/2)k_A^2/I_{\max}$	$\beta_4 = -(\omega_3/2 + \omega_6/2)k_B^2/I_{\max}$
$\beta_5 = \omega_4 k_{AB} - \omega_5 k_A - \omega_6 k_B$	$\beta_6 = \omega_5 k_A^2/I_{\max}$	$\beta_7 = \omega_6 k_B^2/I_{\max}$	$\beta_8 = -(2\omega_4 k_{AB}^2 + \omega_5 k_A^2 + \omega_6 k_B^2)/(2I_{\max})$
$\beta_0 = 1 - k_{dm}$			

Supplementary Note 1: Dataset for comparison study

For the comparison study, two types of input data were employed: 1)a gene expression time series of mouse ESC differentiation as embryoid bodies consisting of 11- time points (0, 6, 12, 18, 24, 36, and 48 hours, and 4, 7, 9, and 14 days) (GEO accession number GSE3231); and 2) binding data of the mouse ESC TFs (Oct4, Sox2, Nanog and Suz12) based on the genome wide ChIP-seq binding peaks from the GSE11724 dataset. We performed peak calling to measure binding targets along genomic sequences using MACS [1]. The binding peaks of ChIP-seq significantly enriched along the promoter regions (-1000 bp ~ +500 bp) were considered as binding occupancy or as regulatory signals of TFs (see Supplementary Data 1).

Supplementary Note 2: Dataset for E2-treated breast cancer

In the first application study, we collected ChIP-seq experiments using breast cancer cell line MCF-7 available GSE26831, GSE29073, GSE32692, GSE32465, GSE14664, GSE19013, GSE54855, GSE40129 from GEO of NCBI, and E-MTAB-223 from ArrayExpress. These data provide binding signals of ERα (ESR1) and breast cancer-related TFs FOXA1, FOXM1, GATA3, CEBPB, JUN, FOS, JUND, as well as cofactors EP300 and CTCF. Similar to Dataset One, the binding peaks of ChIP-seq significantly enriched along the promoter regions (-1000 bp ~ +500 bp) were considered as binding occupancy or regulatory signal of TFs. Time-course gene expression microarray data of estrogen-treated MCF-7 were extracted from accession number E-TABM-742 of ArrayExpress. The 12 time points 0, 1, 2, 4, 6, 8, 12, 16, 20, 24, 28 and 32 hours (h) following the treatment were divided into three stages, T1 (0-2 h), T2 (4-20 h) and T3 (24-32 h). The differential genes with fold difference of expression level at least 1.5 based on the two comparisons between T1 and T2 (T1-T2) or between T2 and T3 (T2-T3) were used as input, respectively. To run LogicTRN, we selected input genes that differentially expressed at least 1.5 fold change difference at T1-T2 or T2-T3 stage in E2-treated breast cancer cells.

Supplementary Note 3: Dataset for hiPSC-derived cardiomyocytes

In the second application study, the conserved binding sites of eight cardiac TFs, MESP1, MEF2C, HAND1, HAND2, GATA4, GATA6, NKX2-5, and TBX5 were considered as the binding targets of TFs. To analyze the conserved target genes, we employed PWMSCAN and FastPval to predict binding sites of the TFs [3, 7]. The genes containing conserved binding sites between human and mouse on the promoters (-2000 ~ +500 bp) are considered as the binding targets of TFs. Time-course gene expression data of hiPSC-derived CM was extracted from GSE35671 of NCBI GEO. The hiPSCs were generated from a human fibroblast cell line via reprogramming and then were differentiated to CM consisting of 0, 3, 7, 10, 14, 20, 28, 35, 45, 60, 90, and 120 days. We divided the 12 time points among 4 stages, T1 (0-3 d), T2 (7-20 d), T3 (28-45 d) and T4 (60-120 d). To run LogicTRN, we selected input genes that differentially expressed at least 2.0 fold changes at the any stage of T1-T2, T2-T3 or T3-T4 in during the hiPSC-derived CM differentiation.

Supplementary Methods
Elaborating the model with a concrete example

Here we elaborate how the model equation can be formulated on a gene regulated by two TFs (p = 2). According to the definitions of the logic OR, AND and NOT, two TFs can form at most six URLs, as shown in Supplementary Table 1, in which each URL is assumed to have a probability of being involved in gene regulation.

Meanwhile, if we assume that a 2-order Taylor expansion can be applied to the nonlinear regulatory function, then there will be 8 composite variables in total, as shown in Supplementary Table 2, in which each composite variable is assumed to have a coefficient in the model equation.

Therefore, the model equation of gene regulation can be written as:

$$\hat{y}_m(t_l) = \beta_1 Y_A + \beta_2 Y_B + \beta_3 Y_A^2 + \beta_4 Y_B^2 + \beta_5 Y_A Y_B + \beta_6 Y_A^2 Y_B + \beta_7 Y_A Y_B^2 + \beta_8 Y_A^2 Y_B^2 + \beta_0 y_m(t_{l-1}) + \varepsilon_l \quad (1)$$

where $\beta_0 = 1 - k_{dm}$ and $l = 2,\cdots,L$, and L is the number of time points. In fact, the model coefficients are functions of kinetic parameters and URLs' probabilities, as showing in Supplementary Table 3.

Calculation of the number of URLs and composite variables
The number of URLs increases drastically with the number of TFs (p), which can be calculated with the formula below:

$$C_P^1 + C_P^2[m_2] + \cdots + C_P^k m_k + \cdots + C_P^P m_P, \text{ where } m_k = 4C_k^1 m_{k-1} - 2(k-1) - N_k^k, \text{ and } m_1 = 1 \quad (2)$$

For instance, the numbers of URLs are 1, 6, 53, 758, 14747, and 351816, respectively, when p is 1, 2, 3, 4, 5 and 6.

Meanwhile, the number of composite variables increases with p *and* n, which can be calculated using the formula of $(n+1)^p - 1$. For instance, in case n is 2, there are 2, 8, 26, 80, 242, and 728 composite variables, when p is 1, 2, 3, 4, 5 and 6, respectively.

Estimation of the confidence value of a regulatory logic
Let $y_m(t_l)$ be the expression level of a gene at time t_l, where $l = 1,\cdots,L$. The model coefficients can be estimated by solving a least square regression problem:

$$(\boldsymbol{\beta}, k_{dm}) = \arg\min_{\boldsymbol{\beta}, k_{dm}} \left\{ \sum_{l=2}^{L} \left(\hat{y}_m(t_l) - y_m(t_l) \right)^2 \right\} \quad (3)$$

To prevent the problem of overfitting during optimization, we can adopt a L1 regularization approach, LASSO. After LASSO regression, a Beta matrix of the regression coefficients in different levels of shrinkage will be obtained:

$$\text{Beta} = \begin{bmatrix} b_{11} & b_{12} & \cdots & b_{1N_Z} \\ b_{21} & b_{22} & & \\ \vdots & & \ddots & \\ b_{M1} & & & b_{MN_Z} \end{bmatrix} \quad (4)$$

in which N_Z is the number of composite variables, M is either $N_Z - 1$ or $L - 2$ whichever is smaller. Each row of the Beta matrix represents a solution at a particular shrinkage level, which increases from 0 (corresponding to the first row of the matrix) to 1 (corresponding to the last row).

The shrinkage parameter determines the sparsity of the coefficients in a row. By comparing non-zero coefficients with the signatures of URLs in Supplementary Data 3, we can obtain the confidence value of each URL.

Let $\mathbf{b}_i$ represents the ith row of the Beta matrix, which is

$$\mathbf{b}_i = [b_{i1}, \cdots, b_{iN_Z}] \tag{5}$$

To moderate the influence of large values, $\mathbf{b}_i$ is normalized to the range $[0, 1]$ by using a sigmoid function as below:

$$b'_{ij} = \frac{1 - \exp(-|b_{ij}/\sigma_i|)}{1 + \exp(-|b_{ij}/\sigma_i|)} \tag{6}$$

where σ_i is the standard deviation of the non-zero elements in $\mathbf{b}_i$. The normalized coefficient, b'_{ij} represents the confidence of keeping the jth composite variable in the model.

Let $\mathbf{v}_k = [v_{k1}, \cdots, v_{kN_Z}]$ be the signature values of the kth logic, in which v_{kj} is either 1 or 0 representing whether the kth logic should employ the jth composite variable or not. Here we estimate the matching probability (MP) for the jth position of $\mathbf{b}_i$ and $\mathbf{v}_k$:

$$mp_j^{(i,k)} = (1 - v_{kj}) \cdot (1 - b'_{ij}) + v_{kj} \cdot b'_{ij} \tag{7}$$

where $mp_j^{(i,k)} = 1$ means a perfect match between v_{kj} and b'_{ij}, while $mp_j^{(i,k)} = 0$ means no matching. The overall MP between $\mathbf{b}_i$ and $\mathbf{v}_k$ thus represents the confidence of keeping the kth logic in logic prediction, which can be calculated as:

$$c_k^{(i)} = \prod_{j=1}^{N_Z} f\left(mp_j^{(i,k)}\right) \tag{8}$$

where $f(\cdot)$ is defined as:

$$f(x) = \begin{cases} x, & if\ x \geq \theta \\ \rho, & if\ x < \theta \end{cases} \tag{9}$$

θ is a threshold between 0 and 1, which is set to be 0.7. ρ is a penalty parameter when there is a mismatch between $\mathbf{b}_i$ and $\mathbf{v}_k$, which is set to be 0.3.

To extract more information from the Beta matrix, we recommend to pick multiple rows in the matrix to estimate the confidence of a URL. Suppose the rows from r_1 to r_2 of the Beta matrix are picked, then overall confidence of the kth URL can be calculated as:

$$c_k = \frac{\sum_{i=r_i}^{r_2} p_k^{(i)}}{\max\limits_{k \in [1, N_R]} (\sum_{i=r_1}^{r_2} p_k^{(i)})} \tag{10}$$

where $k \in [1, N_R]$, and N_R is the number of URLs. The URL with largest confidence value is considered as the dominant logic. Once the dominant logic is determined, both the regulator TFs and their interactive relationships are known.

Estimating the TF-DNA occupancy

In addition to dynamic gene expression data, LogicTRN requires the input of TF-DNA binding occupancy, which is not directly available in real-world dataset. Here we adopted a biologically plausible approach to estimate the TF-DNA occupancy. By definition, TF-DNA occupancy is the probability that the binding sites are occupied by a TF, which can be described as a function of two parameters: concentration of the TF, and the stability of the TF-DNA complex. TF-DNA occupancy is often expressed as:

$$Y = \frac{K_r P_0}{D_n + K_r P_0} \tag{11}$$

in which P_0 is the TF concentration, K_r is the equilibrium constant, D_n is the concentration of the non-specific non-occupied sites. The function of TF-DNA occupancy has three properties: (a) If P_0 is zero then Y will be zero; (b) if P_0 is too large, then Y will approach 1; and (c) when P_0 is in the range between zero to a moderate number, Y is approximately linear to P_0. According to these characteristics, function (11) can be approximated with:

$$Y = 1 - \exp(-\frac{K_r}{D_n} P_0) \tag{12}$$

Note when a 2-order Taylor expansion is applied to (11) and (12), they become $Y = K_r P_0 / D_n - (K_r P_0 / D_n)^2$, and $Y = K_r P_0 / D_n - (K_r P_0 / D_n)^2 / 2$, respectively. Consequently, (12) is a good approximation to (11). Using (12) can extremely simplify the mathematical expression.

One obstacle in estimating the occupancy is that the value of P_0, K_r and D_n are normally not available in most of studies. Due to the fact that TF binding domains are normally conserved in terms of sequence and 3D structure [8]. We can reasonably assume that the binding domains in different protein subunits encoded by a particular TF gene are conserved. In other word, the amount of protein binding domains should be proportionally correlated with the expression of the TF gene. Let y_{TF} be the TF gene expression, k_T be the translation efficiency, and τ is the translational delay, then the concentration of TF protein at time t can be expressed as $k_T y_{TF}(t-\tau)$. Moreover, a binding domain can potentially bind to different gene sites. The binding occupancy between a specific domain D_i (on protein) and a specific gene site S_j (on DNA), $Y_{D_i S_j}$, can be expressed as following:

$$Y_{D_i S_j}(t) = 1 - \exp\left(-K_{D_i S_j} \cdot a \cdot y_{TF}(t-\tau)\right) \tag{13}$$

where $K_{D_i S_j}$ is the domain-site binding affinity between the ith domain and the jth site, and $a = k_T / D_n$. If we assume that domain D_i can bind to N_S sites of a target gene, then the overall occupancy of domain D_i to the target gene can be expressed as:

$$Y_{D_i}(t) = 1 - \prod_{j=1}^{N_S}\left(1 - Y_{D_i S_j}(t)\right) \tag{14}$$

Substituting (13) into (14), we get

$$Y_{D_i}(t) = 1 - a \cdot y_{TF}(t-\tau) \cdot \left(\exp\left(-\sum_{j=1}^{N_S} K_{D_i S_j} \right)\right) \tag{15}$$

Moreover, a TF protein may contain multiple binding domains. Therefore, the overall occupancy of this TF protein to its target gene can be expressed as:

$$Y(t) = 1 - \prod_{i=1}^{N_B} \left(1 - Y_{D_i}(t)\right) \tag{16}$$

where N_B is the number of binding domains on the TF. Substituting (15) into (16), we get:

$$Y(t) = 1 - a \cdot y_{TF}(t-\tau) \cdot \exp\left(\sum_{i=1}^{N_B} \sum_{j=1}^{N_S} K_{D_i S_j} \right) \tag{17}$$

Here we estimate a as a whole for TF by assuming that majority of the genes will be expressed in a normal range. That is, in a biological process, only a small fraction of genes (e.g. less than 5%) can reach its saturated TF-DNA binding status (e.g. TF-DNA binding occupancy up to 0.95). Let $Y_{uv}(t)$ represent the binding occupancy of the TF u to the Gene v. $Y_{uv}(t)$ constitutes a three dimensional matrix regarding to u, v, and t. The coefficient a is then adjusted so that only 5% of the element in the $Y_{uv}(t)$ matrix can reach to 0.95 or above.

Algorithm of implementing LogicTRN

The implementation algorithm of LogicTRN is given as below:

Step one: For a target gene, preliminarily choose the candidate TFs according to literature or database;

Step two: Enumerate all available URLs and all the composite variables, to construct the signatures of URLs;

Step three: Compute the dynamic TF-DNA binding occupancies using dynamic expression data of the target gene and the TF-DNA binding signal;

Step four: Form a series of model equations of pairwise-logic model using dynamic gene expression data and TF-DNA occupancies;

Step five: Obtain the Beta matrix of regression coefficients using LASSO;

Step six: Compute the confidence values of all the URLs in regulating the target gene;

Step seven: Determine the dominant URL which includes both the regulator TFs and the logic;

Step eight: Repeat Step one to seven for each target gene, so as to reconstruct the TRN of the biological process.

Enrichment analysis of regulatory logics

The hypergeometric distribution test was used to evaluate whether the identified logics by LogicTRN and the target gene sets are significantly enriched among the whole differentially expressed gene set. The significance was measured by the p value defined as follows:

$$P_{val} = 1 - \sum_{i=0}^{N_{ovp}-1} \frac{\binom{N_{reg}}{i}\binom{N_{all} - N_{reg}}{N_{Deg} - i}}{\binom{N_{all}}{N_{\deg}}} \tag{18}$$

where N_{all} is the total number of genes that are associated with the gene expression dataset, $N_{\deg}$ is the number of genes which belongs to the set of differentially expressed genes, N_{reg} is the number of genes regulated by a logic, and N_{ovp} is the number of overlapped genes in both lists of $N_{\deg}$ and N_{reg}. p values were then corrected by FDR to obtain the FDR-p values.

Negative control analysis of regulatory logics

We constructed negative controls for TFs involved in gene regulation by collecting the logics that were absent from the enriched logics or URLs but have the same TFs involved. The enrichment of

the target genes among the differentially expressed genes was evaluated. The p values of the enrichment were then transformed to negative 10-based logarithm. We tested the predicted logics in human breast cancer and hiPSC-CM, the p values between enriched URLs and negative controls were calculated, respectively by the two-tail students test.

Supplementary References

1. Zhang Y, *et al*. Model-based analysis of ChIP-Seq (MACS). *Genome biology* **9**, R137 (2008).

2. Guan D, *et al*. PTHGRN: unraveling post-translational hierarchical gene regulatory networks using PPI, ChIP-seq and gene expression data. *Nucleic acids research* **42**, W130-136 (2014).

3. Levy S, Hannenhalli S. Identification of transcription factor binding sites in the human genome sequence. *Mammalian genome : official journal of the International Mammalian Genome Society* **13**, 510-514 (2002).

4. Li MJ, Sham PC, Wang JW. FastPval: a fast and memory efficient program to calculate very low P-values from empirical distribution. *Bioinformatics* **26**, 2897-2899 (2010).

5. Yan B, *et al*. Unraveling regulatory programs for NF-kappaB, p53 and microRNAs in head and neck squamous cell carcinoma. *PloS one* **8**, e73656 (2013).

6. Wang P, *et al*. ChIP-Array 2: integrating multiple omics data to construct gene regulatory networks. *Nucleic acids research* **43**, W264-269 (2015).

7. Li MJ, Sham PC, Wang J. FastPval: a fast and memory efficient program to calculate very low P-values from empirical distribution. *Bioinformatics* **26**, 2897-2899 (2010).

8. Nitta KR, *et al*. Conservation of transcription factor binding specificities across 600 million years of bilateria evolution. *eLife* **4**, (2015).
