# Supplementary material for: An integrative method to decode regulatory logics in gene transcription
Source: Nat Commun. 2017 Oct 19;8:1044. doi: 10.1038/s41467-017-01193-0 (PMC5715098; doi:10.1038/s41467-017-01193-0)
Supplement: Supplementary file 3 — Supplementary Files [file 41467_2017_1193_MOESM3_ESM.pdf]

Description of Supplementary Files

File name: Supplementary Data 1
Description: Signature of Unique Regulatory Logics (URLs) in simulation study

File name: Supplementary Data 2
Description: Binding target genes of mouse ESC regulators based on ChIP-seq peak calling on promoters

File name: Supplementary Data 3
Description: Comparison of LogicTRN with COGRIM, APG, NCA, and PTHGRN in identifying target genes of mouse ESC TFs (Oct4/Pou5fl, Sox2, Nanog and Suz12). I - Lists of known (validated) ChIP-seq binding target of mouse ESC TFs, II - positive set, III - negative set, IV - Lists of predicted target genes of mouse ESC TFs using 5 algorithms (Score represent decision for testing AUC and AUPR, 1=maximal, 0=minimal)

File name: Supplementary Data 4
Description: Predicted regulatory logics in breast cancer cell

File name: Supplementary Data 5
Description: Predicted regulatory logics in hiPSC-derived cardiomyocytes
